# Modeling Recursive RNA Interference

**DOI:** 10.1371/journal.pcbi.1000183

**Published:** 2008-09-19

**Authors:** Wallace F. Marshall

**Affiliations:** Department of Biochemistry and Biophysics, Integrative Program in Quantitative Biology, University of California San Francisco, San Francisco, California, United States of America; University of Queensland, Australia

## Abstract

An important application of the RNA interference (RNAi) pathway is its use as a small RNA-based regulatory system commonly exploited to suppress expression of target genes to test their function in vivo. In several published experiments, RNAi has been used to inactivate components of the RNAi pathway itself, a procedure termed recursive RNAi in this report. The theoretical basis of recursive RNAi is unclear since the procedure could potentially be self-defeating, and in practice the effectiveness of recursive RNAi in published experiments is highly variable. A mathematical model for recursive RNAi was developed and used to investigate the range of conditions under which the procedure should be effective. The model predicts that the effectiveness of recursive RNAi is strongly dependent on the efficacy of RNAi at knocking down target gene expression. This efficacy is known to vary highly between different cell types, and comparison of the model predictions to published experimental data suggests that variation in RNAi efficacy may be the main cause of discrepancies between published recursive RNAi experiments in different organisms. The model suggests potential ways to optimize the effectiveness of recursive RNAi both for screening of RNAi components as well as for improved temporal control of gene expression in switch off–switch on experiments.

## Author Summary

RNA interference is a gene regulatory system in which small RNA molecules turn off genes that have similar sequences to the small RNAs. This has become a powerful tool because a researcher can use RNA interference to turn off any gene of interest in order to test its function. There is great interest in identifying the genes required for the RNA interference pathway, and one approach to identifying such genes has been to use RNA interference to turn off potential RNA interference genes and to ask whether RNA interference still functions when these genes are turned off. The goal of our report is to ask how it is possible for RNA interference to turn itself off, using a mathematical model of the system. The results show that RNA interference cannot turn itself off if the RNA interference pathway is too effective to start with, so that experiments in which RNA interference acts on itself will only work in systems having a low efficiency. The results of our model suggest possible ways to improve the self-inactivation of RNA interference.

## Introduction

RNA interference (RNAi) is an RNA-mediated pathway of gene silencing mediated by small RNA molecules [1],[2]. During RNAi, introduction of double-stranded RNA (dsRNA) encoding a sub-sequence of a gene leads to reduction in expression of the corresponding gene. The heart of the RNAi process involves two key steps. First, the dsRNA is cleaved into small RNA fragments by an enzyme called Dicer, and then these small fragments are used as a template by a complex called RISC which identifies matching sequences in target messages and leads to their degradation.

RNAi technology has emerged as a powerful tool for artificially controlling gene expression, but it only works because cells have evolved small RNA based regulatory pathways in the first place. Natural regulatory pathways taking advantage of small RNAs include not only classical RNAi, which probably acts in host defense against viruses and transposons, but also microRNA-based (miRNA) regulatory pathways that regulate endogenous genes [3]. It is interesting to speculate that such pathways may have evolved in part because of unique aspects of regulation mediated by RNA. Compared to more classical regulatory networks based on transcription factors or kinases, the signal-processing properties of small RNA-based regulatory systems have not been extensively investigated at a theoretical level. One advantage of having a theoretical understanding of such pathways is that one could potentially predict the performance and response of systems that have been altered in defined ways, thus facilitating a “synthetic biology” of small RNA-mediated regulatory circuits [4],[5]. For a more short-term application, one might hope that a predictive level of understanding of RNAi pathway behavior could allow improved design of experiments using RNAi as a tool. In this report the RNAi system is explored theoretically by considering its behavior following addition of an artificial negative feedback loop.

It is well known in electronics that when the output of a circuit is fed back into one of its inputs, the resulting closed-loop circuit can have dramatically different behaviors than the open-loop circuit before the feedback loop was added. A key challenge for systems biology is to be able to predict the effect of feedback loops on biological circuits, either naturally occurring feedback or synthetic feedback produced by adding new linkages from output to input [6]. In the case of naturally occurring small RNA-mediated regulatory loops based on micro-RNAs, feedback loops are sometimes seen in which components of the RNAi/miRNA machinery such as Dicer or Argonaute are themselves targets of miRNA-mediated inhibition [7],[8]. Being able to quantitatively or even qualitatively predict the effect of such feedback linkages would therefore seem crucial to developing a circuit theory for small RNA based signaling [9].

In the case of the RNAi pathway, synthetic feedback loops have been constructed by workers attempting to use RNAi to turn off the RNAi pathway. This is done simply by adding dsRNA molecules that target genes encoding components of the RNAi machinery. In such a situation, the feedback can be considered as arising from the output of the RNAi machinery (that is, degradation of target message) being applied as an input to the system in the form of message encoding RNAi components. This “recursive” RNAi has been used in genome-wide screens to discover new RNAi components [10]–[14]. In such screens, a reporter gene such as green fluorescent protein (GFP) or luciferase is silenced by RNAi, and then reporter activity is measured in the presence of a second dsRNA molecule targeting a candidate gene. Increased reporter expression indicates that the candidate gene is involved in the RNAi process. By using libraries of dsRNA molecules corresponding to all predicted genes in the genome, it is in principle possible to identify all components of the RNAi machinery. In order for screens of this type to be successful, the reporter activity must be significantly increased over the level seen when the reporter alone is targeted.

Recursive RNAi has also been used as a way to reactivate genes previously silenced by RNAi. Such “switch-off/switch-on” experiments employ a procedure in which a dsRNA is introduced targeting a gene of interest, and then, following a period of inactivation, the RNAi is alleviated by adding a second dsRNA that targets the RNAi machinery itself [15]. This allows temporal control of gene expression during animal development, and has the advantage that it can be applied to any gene without having to engineer new inducible constructs for each experiment. In order for switch-off/switch-on experiments to work, the level of restoration of the targeted gene must be enough to restore approximately normal gene function. For strictly recessive genes this would probably require restoration to approximately half normal levels, while for haploinsufficient genes it would require a greater degree of restoration, to near wild-type levels. Recursive RNAi can thus potentially be a very powerful tool both for studying RNAi itself and also for controlling gene expression during development, provided a sufficient level of restoration can be achieved once the RNAi machinery is targeted.

Despite the great potential of recursive RNAi, and the multiple published successes of the method, one cannot help but feel that the use of RNAi to inactive RNAi seems potentially self-defeating. Specifically, one might imagine that as the pathway is shut down, its ability to further shut itself down would be reduced, resulting potentially in a restoration of activity. Recursive RNAi presents the same difficulty as attempting to commit suicide by holding one's breath—even if one could hold one's breath to the point of passing out, the unconscious patient would at that point begin breathing again. The quantitative question thus arises as to whether introduction of recursive RNAi would provide a restoration of gene expression level that would be measurable or detectable relative to control levels. Indeed, in actual practice recursive RNAi doesn't always work. For instance, although some studies have reported that RNAi of genes encoding Dicer protein restores reporter gene expression [16], other studies failed to observe significant restoration following RNAi of Dicer [11]. One possible explanation for the variability in results between different systems is the efficacy of RNAi at knocking down gene expression. Some cell types such as S2 cells can achieve extremely high levels of knockdown to a few percent of wild-type expression levels [11] while other systems such as C. elegans RNAi-by-feeding seem to produce a more moderate degree of knockdown. Might such variation make recursive RNAi possible in some systems and impossible in others? This report investigates the conditions under which recursive RNAi can be effective, by constructing a mathematical model for recursive RNAi and predicting how its performance varies as a function of the efficacy of RNAi in a given system. The main prediction of the model is that increasing the efficacy of RNAi-mediated knockdown should make recursive RNAi less efficient and potentially impossible.

## Results

### Relative Susceptibility of RNAi Components to RNAi

**Figure 1:**
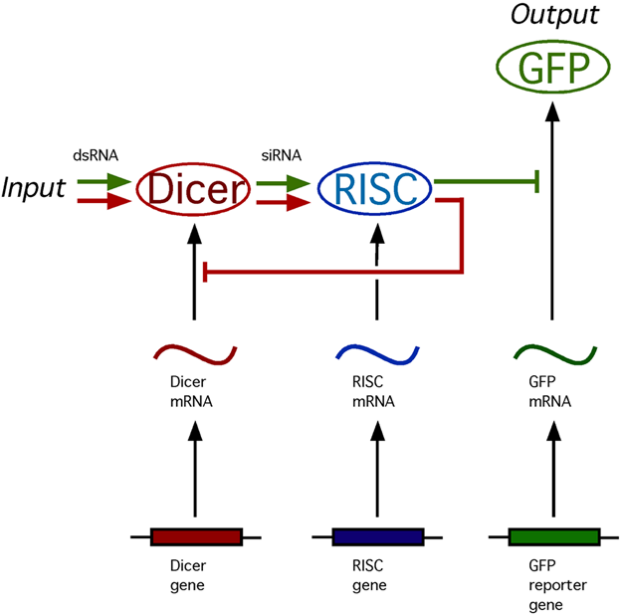
Diagram of recursive RNAi circuit. RNAi takes place in two steps. Following input to the system of a double stranded RNA precursor (dsRNA), Dicer chops the dsRNA into small interfering RNA molecules (siRNA) which are then used by the RISC complex to direct cleavage of target messages. At the same time, genes encoding RNAi machinery as well as the reporter construct (in this case GFP) are transcribed into mRNA and then translated into protein (indicated by ovals in the diagram). RNAi repressed gene expression by providing an extra decay pathway for the targeted message, so that rather than being translated into protein the message was destroyed. In recursive RNAi, two dsRNA molecules are provided as input, one directed against the reporter gene and the other directed against a gene encoding part of the RNAi machinery itself. The measurable output of the system is the level of reporter protein (GFP).

The RNAi pathway upon which the model is based is shown schematically in Figure 1, and based on this diagram a model is presented in the Materials and Methods section below. Within the model, the steady-state behavior of the system is specified by a single parameter, γ, which determines the overall effectiveness of RNAi in a particular cell type. RNAi efficacy can be expressed in terms of the fold-knockdown achievable, that is, the ratio of expression level prior to RNAi relative to the expression level following RNAi. For instance, a gene whose expression is reduced to one half its normal level by RNAi would show a fold-knockdown of 2-fold. As derived in Materials and Methods, the fold knockdown predicted for a reporter gene such as GFP or luciferase, in the absence of any additional RNAi targeting Dicer or RISC, would be described in terms of an RNAi efficacy parameter γ according to the following equation:
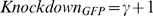
(1)


Thus the parameter γ determines the efficacy of RNAi system, with larger γ indicating more extensive knockdown of gene expression. As described in Materials and Methods, and summarized in Table 1, this parameter depends on all of the individual parameters of the detailed model, such as the catalytic rate constants of Dicer, the rate of mRNA degradation, etc. Many of the individual rate constants and parameters that contribute to γ may be extremely difficult to measure. In contrast, because of the simple relation between fold-knockdown and the value of the parameter γ this parameter is experimentally measurable simply by quantifying reporter level before and after RNAi. Typical values for γ are in the range 2–200. Moreover, because the steady-state behavior of the system depends only on this one parameter γ, for many purposes it may not be critical to know the values of the detailed parameters given in Table 1, as long as one knows the value of the aggregate RNAi efficacy parameter γ. In this paper the parameter γ is generally imagined to vary over the range 1–200. The variations of the detailed parameters listed in Table 1 are not considered individually because their only effect on the model behavior is through their influence on the value of γ. A second model parameter β plays a role in determining the time-scale over which RNAi knocks down its targets, and is therefore also directly experimentally measurable. Because β has no effect on the steady-state level of knockdown, this parameter will not be considered except when the transient behavior of the system is analyzed. β and γ are the only two adjustable parameters of the model. Both parameters are phenomenological and easily measurable using standard methods of quantifying RNAi efficiency, but both parameters can also be defined in terms of detailed mechanistic parameters such as protein turnover rate, as described in Materials and Methods.

**Table 1:** Parameters of RNAi model.

Mechanistic parameters	
r_ds_	siRNA degradation rate constant
r_dp_	Protein degradation rate constant
r_dm_	mRNA degradation rate constant
r_x_	Translation rate constant
r_t_	Transcription rate constant
k_catD_	Catalytic rate constant for Dicer-mediated siRNA production
k_catR_	Catalytic rate constant for RISC-mediated target degradation
K_DR_	Dissociation constant for siRNA with RISC
Lumped parameters	
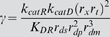	RNAi efficacy parameter
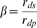	RNAi settling time parameter

The first set of parameters describes the rate constants of the individual steps in the overall reaction scheme shown in Figure 1. As derived in Materials and Methods, these mechanistic parameters dictate behavior only through their combined effect on two lumped parameters, gamma and beta, which determine the level of knockdown achievable by RNAi and the time required to achieve knockdown. Every mechanistic parameter contributes to at least one of the two lumped parameters. The complete range of behavior of the model can be obtained by varying just γ and β; thus, specific values of the detailed mechanistic parameters are not considered.

**Figure 2:**
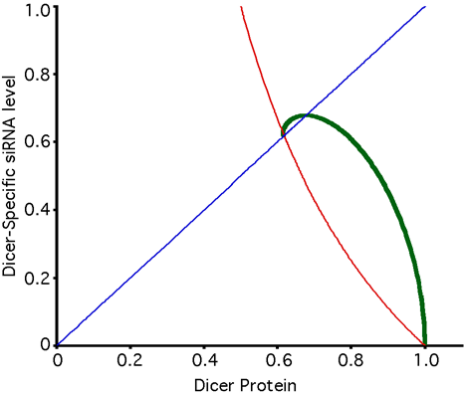
Phase plane diagram of recursive RNAi of Dicer. Phase plane diagram of Dicer RNAi showing the nullclines for which the rate of change of either the siRNA (in blue) or Dicer protein (in red) equals zero. The two curves only intersect for one set of values, indicating a unique steady-state solution. The transient solution starting from an initial condition of normal dicer level and zero Dicer-directed siRNA is plotted in green, obtained by numerical integration. This simulation was conducted with an RNAi efficacy parameter gamma equal to 1.

When dsRNA is introduced to target a gene encoding a component of Dicer, the system stably attains a new steady state in which the level of the targeted Dicer-specific protein is partially reduced (Figure 2). As detailed in Materials and Methods, the model predicts that the inherent susceptibility of Dicer to knockdown by RNAi differs from that of a reporter gene, with the fold-knockdown for Dicer given by
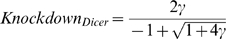
(2)


The same equation is predicted to describe the susceptibility of RISC when it is targeted by recursive RNAi, indicating the two parts of the RNAi pathway have similar susceptibility to RNAi mediated knockdown.

It is perhaps of interest to note that, for γ = 1, corresponding to a two-fold knockdown of the reporter, the fold knockdown predicted for Dicer from Equation 2 is 2/(−1+√5). This is the famous “Golden Ratio”, known since Greek antiquity to arise in situations involving self-similarity and recursion.

The major biological significance of Equations 1 and 2 is that genes encoding components of the Dicer and RISC complexes are inherently less susceptible to RNAi knockdown compared to genes not involved in the RNAi pathway. This differential susceptibility raises questions about detectability of recursive RNAi. Would reporter gene expression be restored significantly if Dicer was simultaneously targeted? As detailed in Materials and Methods, the model predicts RNAi-mediated reporter knockdown in the presence of RNAi targeting components of Dicer (or of RISC—the equation ends up being the same) to be:
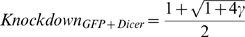
(3)


**Figure 3:**
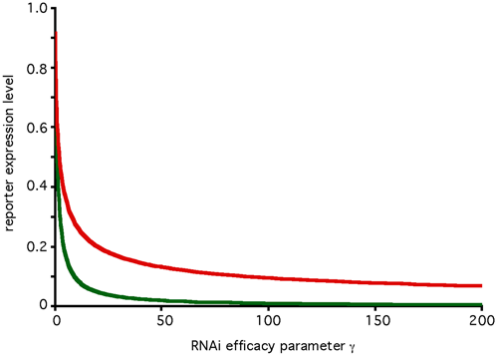
Restoration of reporter expression during recursive RNAi. Predicted reporter expression in the presence (red curve) and absence (green curve) of Dicer-specific RNAi. As the two curves approach each other, the restoration becomes more difficult to detect.

Figure 3 graphs the predicted expression levels of a reporter gene targeted by RNAi in the presence (Equation 3) or absence (Equation 1) of recursive RNAi targeting Dicer, plotted as a function of the underlying RNAi efficacy in the system. Clearly, the level of reporter gene recovery depends on the efficiency of RNAi in the system, such that more effective RNAi predicts less recovery of reporter expression. As γ becomes large (i.e. knockdown is very efficient), the reporter expression levels obtained with and without recursive RNAi gradually approach each other, making the effect potentially very hard to detect over measurement noise.

### Comparison with Experimental Results

These results can reconcile the apparent disagreement in the literature concerning the efficacy of recursive RNAi of Dicer, because the variation in RNAi efficacy (as described by parameter γ) between cell types and organisms should produce predictable variation in restoration (Figure 3). Comparison of the predicted restoration to published data reveals a remarkably good match. Bernstein et al [16] describe experiments in which a GFP reporter reduced to 15% of control levels by RNAi is restored to 40% of control levels when Dicer is simultaneously targeted. From Equation 1, 15% knockdown implies γ = 5.5, from which Equation 3 predicts restoration to 35% of control levels, consistent with the experiments. In a different cell type (human HEK293 cells) Schmitter et al [17] found that RNAi directed against a luciferase reporter knocked expression down to 45% of normal levels, and simultaneous targeting of Argonaute-2 restored expression to 60% of pre-RNAi levels. From Equation 1, reporter knockdown to 45% implies γ = 1.2, hence Equation 3 predicts restoration to 60% of control levels, exactly as observed. In these cases a moderately effective RNAi system yields substantial restoration during recursive RNAi, as predicted. In a contrasting example, Dorner et al. [11] describe a highly effective RNAi system in which the reporter was knocked down to 0.5% of control levels, corresponding to γ = 200. Equation 3 predicts Dicer-specific RNAi should restore reporter expression only to 7% of controls, a relatively small recovery. Consistent with this prediction, Dorner et al. found that RNAi targeting a number of RNAi components such as Dicer-2 and R2D2 only increased reporter expression slightly to a few percent of control levels. A similar low level of restoration of reporter activity was reported in a separate study of RNAi of Dicer-2 in S2 cells [18]. In an even more extreme case, Hoa et al. [19] performed recursive RNAi in mosquito cells for which RNAi of luciferase knocks down the reporter 4000-fold. In this extremely efficient RNAi system, the authors found that targeting of Dicer only restored the luciferase reporter to 2% of control levels. A 4000-fold knockdown implies γ = 3999, from which Equation 3 predicts a restoration of the reporter to 1.6% of control levels, again consistent with the observed level of restoration. These results suggest that poor restoration by recursive RNAi is likely to be a common feature of highly efficient RNAi systems. Dorner et al. [11] concluded in their study that most of the RNAi machinery genes tested in their experiments were not susceptible to RNAi. However, the model given here suggests the experiments were, in fact, effective, but due to the inherently self-limiting nature of recursive RNAi at high γ, the extent of recovery was simply not very large. The differences in performance between different systems are consistent with the predictions of the model for different values of gamma, but it is impossible to rule out that some of the differences could be due to differences in targeting sequences for the reporter versus for the RNAi machinery (a point to be discussed further below).

The predictions of this model regarding restoration achievable by recursive RNAi of Dicer only apply to experiments in which Dicer is targeted by addition of shRNA or other forms of dsRNA, and the limitation on knockdown is a result of the requirement for Dicer activity to generate siRNA against itself. If Dicer is targeted by directly by introduction of siRNA, then the model might predict a dramatically increased level of restoration since in this situation Dicer-mediated production of siRNA would no longer be required for its own knockdown. Consistent with this, experiments in which Dicer is targeted directly by exogenously introduced siRNA molecules show almost complete restoration of reporter activity [20]. On the other hand, dynamics of the system might be significantly different because while Dicer is not required to produce exogenously added siRNA, it may still be involved in loading these siRNA molecules into the RISC complex [21].

It is to be noted that different siRNA molecules can show extremely large differences in targeting efficiency [22]–[28] and unless the targeting efficiency of each construct is known, it is impossible to compare quantitative results between different constructs and systems, let alone compare a theoretical model with experimental data. Thus, the comparisons presented here should be viewed as showing a qualitative similarity in overall trends, with precise numerical equivalence being impossible to assess until targeting efficiencies are measured for each experiment.

### Optimization of Recursive RNAi Experimental Design

The foregoing results suggest that the effectiveness of recursive RNAi could be improved by reducing the effectiveness of RNAi, for example using mutant backgrounds with partial defects in one or more RNAi components. To optimize the design of recursive RNAi experiments, one approach is to define a figure of merit to describe restoration of reporter activity (see Materials and Methods) and then attempt to maximize its value. A figure of merit can be defined by the relative restoration ratio, R, which is the reporter-specific RNAi-mediated decrease in reporter level in the presence of Dicer RNAi divided by the decrease seen in the absence of Dicer RNAi.

**Figure 4:**
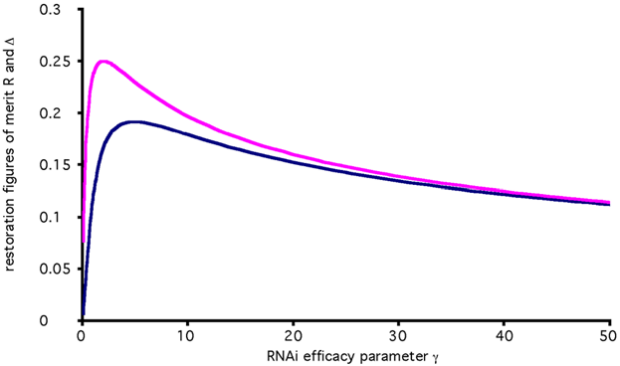
Optimization of restoration. Figures of merit describing restoration efficiency plotted as a function of RNAi efficacy parameter gamma. Pink curve plots the relative restoration ratio R which measures the ratio of restoration relative to the initial level of knockdown. Larger R indicates that gene expression is restored to a level closer to its normal expression level in the absence of any RNAi, as required for switch-off/switch-on experiments [15]. Maximum value of R is 0.25 which occurs for γ = 2, corresponding to a system in which RNAi knocks down gene expression only three-fold. Blue curve plots the normalized absolute restoration Δ which is the reporter level during recursive RNAi minus the reporter level without recursive RNAi, expressed in units normalized by the pre-RNAi expression level of the reporter. Larger values indicate more easily detected restoration. Both curves show a peak, indicating optimal performance, at comparatively low values of gamma.

Figure 4 plots the value of R as a function of the RNAi efficacy parameter γ. It is easy to show that the restoration is maximal when γ equals 2, which corresponds to a 3-fold reduction in reporter level. As overall RNAi efficacy increases past this point, the level of reporter gene restoration achievable by RNAi of RNAi decreases, in other words, the effect of recursive RNAi becomes more difficult to detect.

An alternative figure of merit that may be more appropriate for certain types of screening experiments is the normalized absolute difference Δ between reporter levels with and without recursive RNAi of Dicer (as described in Materials and Methods). As shown in Figure 4, this figure of merit also predicts that the maximum restoration will occur for low values of γ. Thus, by either criterion, the success of recursive RNAi hinges on avoiding the use of highly efficient systems. This confirms the intuition that recursive RNAi can in fact be self-defeating.

### Transient Behavior

**Figure 5:**
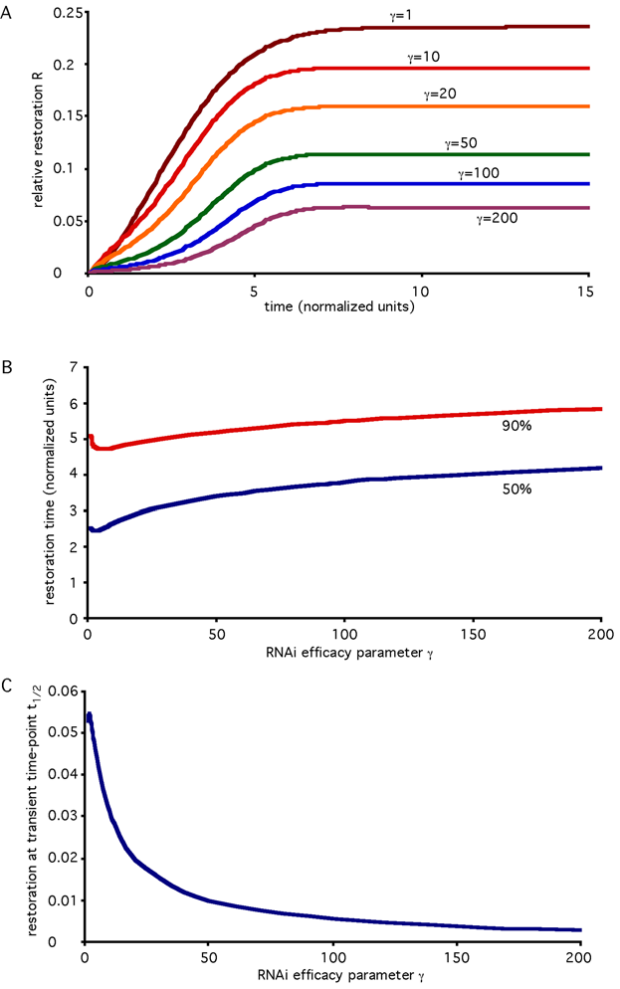
Transient behavior of recursive RNAi. Restoration of reporter levels during recursive RNAi of Dicer determined by numerical simulation. (A) Time-course of restoration of reporter gene level plotted as a function of time following recursive RNAi of Dicer, for different values of the RNAi efficacy parameter gamma. Curves show that even at transient time-points before reaching steady state, restoration is always higher for lower values of gamma. (B) Time required to reach 50% (blue) or 90% (red) of final steady-state restoration value, plotted versus RNAi efficacy parameter. (C) Restoration in reporter seen during recursive RNAi of Dicer at a specific time-point t1/2 defined as the time required for the same reporter gene to be knocked down to half its final level of knockdown in the absence of recursive RNAi. This curve provides a measure of the degree of restoration achieved at a standardized transient time-point, confirming that increasing values of gamma give decreasing restoration, even in the transient case.

The analysis presented thus far treats only the steady-state behavior of the system. In many cases, however, experiments might be conducted before the system has achieved its final steady-state. Would the general conclusion presented above, namely that restoration decreases as RNAi efficacy increases, still hold in a transient condition? Would restoration seen at a transient time-point be greater than that seen at steady state, or less? To answer these questions numerical integration was used to simulate the transient response of the recursive RNAi system following induction of RNAi. Figure 5 illustrates the results of this analysis. First, as illustrated in Figure 5A, the restoration of reporter protein level is a monotonically increasing function of time, so that the restoration achievable at a transient time-point will always be less than that achievable at steady state. This plot shows that there are no unexpected transient dynamics or overshoots, and that rather the system smoothly approaches its steady state. Second, one can note in Figure 5A that the system always reaches its steady-state plateau at roughly the same time, with only a small variation in the time taken to plateau with respect to variation in gamma. This is confirmed in Figure 5B which shows that the time taken to reach a fixed percentage of final restoration depends only weakly on gamma. Indeed, the time to reach 50% or 90% of final restoration varies by less than two-fold when the RNAi efficacy parameter gamma varies by two orders of magnitude. Third, it can be seen in Figure 5A that at all time-points, systems with greater RNAi efficacy (γ) have lower restoration. This is confirmed in Figure 5C, which plots restoration versus gamma at a specific transient time-point defined as the time at which GFP would be knocked down to 50% of its steady-state knockdown level following induction of RNAi. At this transient time-point, the restoration clearly decreases as gamma increases, mirroring the results plotted in Figure 4 for the steady-state behavior. These results indicate that the general conclusions reached about the detectability and effectiveness of recursive RNAi obtained by analytic determination of the steady-state solution also apply to the transient case.

### Recursive RNAi with Unequal Targeting of Reporter and RNAi Component

**Figure 6:**
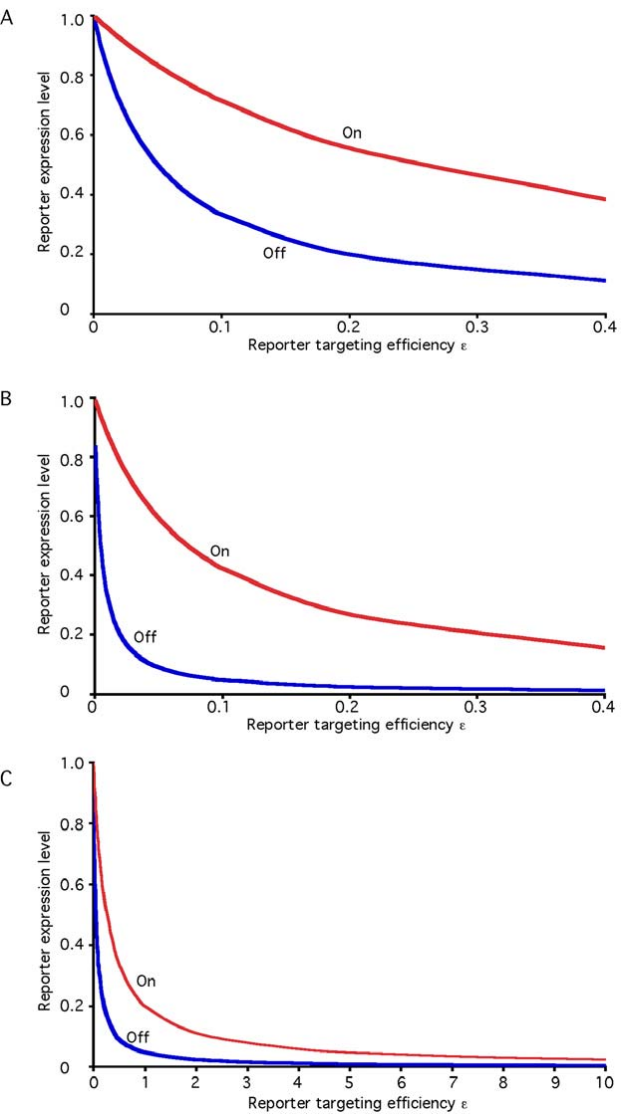
Improving performance of switch-off/switch-on experiments by unequal efficiency of targeting Dicer and Reporter. In each graph the red curve shows reporter level when Dicer is targeted (switch-on state), and the blue curve shows reporter level when Dicer is not targeted (switch-off state). Results are plotted as a function of the efficacy with which the reporter is targeted by the siRNA (defined by parameter epsilon) relative to the efficacy with which Dicer is targeted. The first two graphs show results predicted for different values of overall RNAi efficacy parameter gamma. (A) gamma = 20. (B) gamma = 200. (C) shows results for gamma = 20 over an extended range of targeting efficacy epsilon, with values greater than 1 indicating that the reporter is targeted with higher efficiency than Dicer.

The model described thus far assumes that the target gene (GFP, for instance) is targeted with the same efficiency as the RNAi component gene. It is well known that the efficacy of target degradation caused by a particular siRNA depends significantly on the precise sequence used for targeting [22]–[28]. The effects of unequal targeting of a reporter versus Dicer are derived in Materials and Methods and plotted in Figure 6. The figure shows that as the relative targeting of the reporter is decreased compared to Dicer, the level of restoration can be increased significantly, as indicated by the difference in GFP expression levels with and without recursive RNAi. Figure 6 also shows that the effect becomes more pronounced as γ is increased. In particular, Figure 6 shows that for very efficient RNAi systems (high γ), a more switch-like behavior could be obtained by recursive RNAi provided the targeting of the reporter gene is deliberately made inefficient. This is a prediction that could be tested experimentally by designing a series of dsRNA constructs targeting GFP chosen to span a range of targeting efficiencies, and then measuring the restoration achievable. Figure 6C shows that while restoration can be improved with targeting of the reporter is less efficient, when targeting of the reporter is made more efficient than targeting of the RNAi machinery restoration becomes progressively less efficient. It is thus clearly desirable to tune the relative targeting efficiencies of the two constructs using existing algorithms [22]–[28] in order to decrease the efficacy of reporter targeting relative to the RNAi component that is targeted in recursive RNAi experiments.

### Feedback Confers Reduced Sensitivity to Parameter Variation

A standard reason for employing feedback in electronic circuits is to reduce the sensitivity of the system performance to variations in the operating parameters of components. This is classically seen in operational amplifier circuits which, when connected in a negative feedback mode, produce an amplifier whose gain is almost completely insensitive to variations in the gain of the operational amplifier itself. Gene expression is an inherently noisy process [29], leading to random variation in protein levels for any given gene product. Variation in levels of knockdown has been measured in RNAi experiments and is a significant problem for detectability in genome-wide screens [30],[31]. Might recursive RNAi, by adding a feedback control to the RNAi system, make the system less sensitive to fluctuations in protein levels? In order to investigate whether recursive RNAi might help make the operation of the RNAi system more tolerant to variations in its own components, the sensitivity of Dicer protein levels to variation in the rate of Dicer protein translation was analyzed. Translation of message into protein is often considered a major source of biological noise. Variation in Dicer was chosen for purely hypothetical reasons, there does not appear to be any published data on cell-to-cell variability in protein levels for RNAi components. Sensitivity is defined in this case as the change in Dicer protein level at steady-state caused by a given change in the translation rate of Dicer protein. As derived in Materials and Methods, the ratio of sensitivity in the recursive configuration to that in the open-loop (i.e., non-recursive) configuration is a function of γ, given by the following equation:
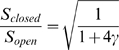
(4)


This equation shows that any change to any parameter of the system that would increase γ will have the effect of making the system less sensitive to variation in the translation rate of Dicer. The same equation can easily be shown to hold for sensitivity to variation in the transcriptional rate of Dicer message. Feedback thus makes RNAi more robust to parameter variation, and the greater the efficacy of RNAi, the greater the improvement in robustness. This may explain why, in some cases, the Dicer gene appears to be under negative feedback control by the miRNA pathway [7].

### Effect of RdRP-Mediated siRNA Amplification

**Figure 7:**
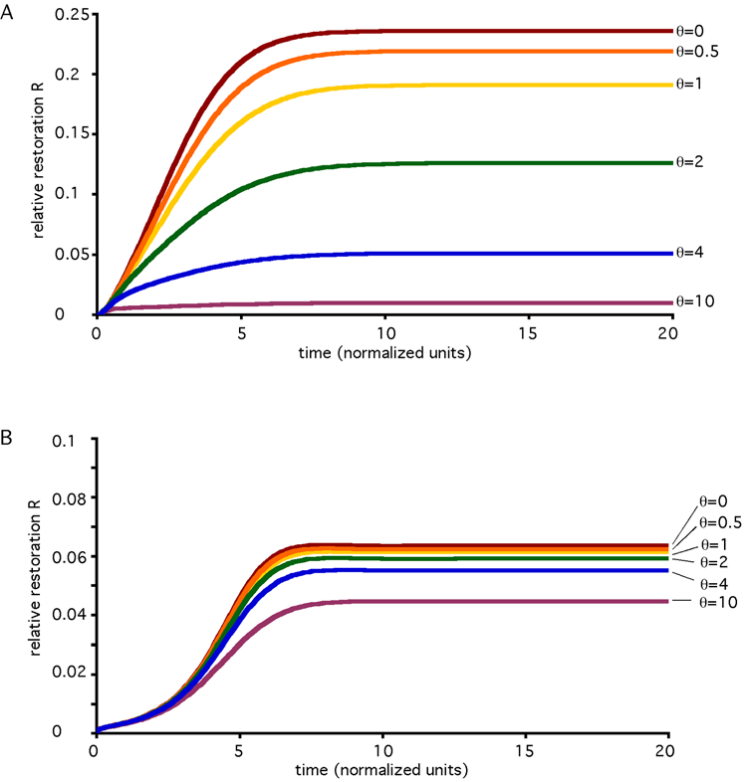
Effect of RdRP-mediated amplification. Each graph shows restoration versus time for numerical simulations of recursive RNAi experiments targeting Dicer. (A) gamma = 1. (B) gamma = 200. Within each graph, results for different values of the amplification efficacy parameter theta are given. Theta is proportional to the number of secondary siRNA molecules produced by RdRP for each targeted mRNA molecule. Modification of model equations to incorporate RdRP activity is described in Materials and Methods.

In some systems, induction of RNAi leads to production of secondary siRNA using the targeted mRNA as a template for an RNA-directed RNA polymerase (RdRP) [32]–[34]. How would this amplification affect the behavior during a recursive RNAi experiment? Figure 7 shows numerical simulation results plotting restoration of a reporter gene for different values of the efficacy of amplification (as described by the parameter theta) simulated at two different values of the RNAi knockdown efficiency parameter gamma. It is clear that increased amplification leads to reduced restoration. This is in keeping with the general conceptual idea that more efficient RNAi, which can be achieved either by higher knockdown efficacy or by increased amplification, leads to decreased restoration in recursive RNAi experiments. Comparing the two panels, it is clear that for any given value of the amplification parameter, lower gamma always leads to better restoration. Thus, the addition of the amplification pathway to the model has no effect on the overall qualitative conclusion that increased efficacy of RNAi leads to decreased restoration.

### Components with Partial Contribution to RNAi Efficacy

**Figure 8:**
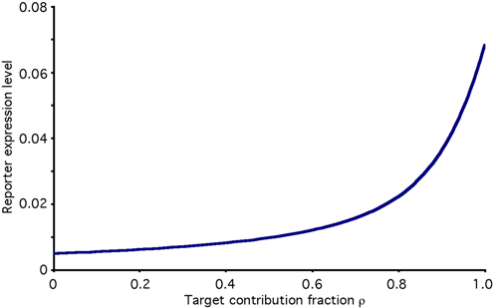
Targeting non-essential components. Graph shows level of reporter expression during recursive RNAi targeting components as a function of the degree to which the component is required for RNAi, indicated by requirement parameter rho. The equation describing this situation is derived in Materials and Methods. Graph plotted for γ = 200.

The analysis presented thus far assumes that if a given RNAi pathway component was knocked down completely, it would result in complete loss of RNAi activity. This effect underlies the potentially self-defeating nature of recursive RNAi. However, only a few proteins of the RNAi pathway appear to be essential core components, with the rest making significant, but not essential, contributions to the process [35]. Even complete knockdown of the non-core components would thus allow some level of RNAi to continue. Would recursive RNAi of such non-core components produce restoration to a different degree than targeting a core component? This question was addressed by modifying the model equations to add a new parameter rho that represents the degree of requirement of a given component for the process of RNAi. A value of ρ = 1 indicates the component is a core component essential for RNAi, while ρ = 0 indicates a component that is not involved in RNAi at all. Low values of rho would also apply for components encoded by multiple redundant gene copies. The expression of a reporter gene in the presence of recursive RNAi is plotted in Figure 8 (based on equations derived in Materials and Methods) as a function of the level of requirement ρ. The result is that recursive targeting of a non-essential component (ρ<1) leads to less restoration than recursive targeting of an essential core component. This implies that variation in the degree of requirement of a given protein for RNAi could be an important source of variation in the level of restoration achievable by recursive RNAi inhibition of different components of the pathway.

### Transient Transfection

There are many ways to introduce dsRNA into cells to activate RNAi. In some cases, the dsRNA is added by soaking or feeding, in others it is expressed by stably integrated constructs. In other cases, however, the dsRNA is expressed as a short hairpin construct contained on a plasmid that is transiently transfected into cells. In this case, the rate of dsRNA production will not be uniform over time because the concentration of plasmid will decrease with first order decay kinetics as the plasmid becomes diluted during cell division.

**Figure 9:**
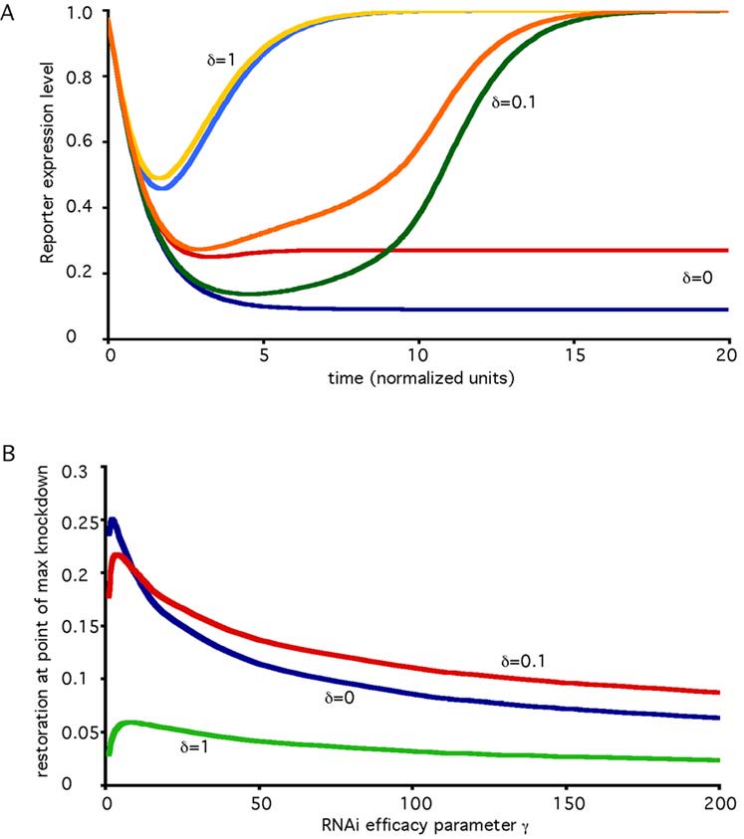
Modeling transient transfection experiments. (A) reporter gene expression levels with and without recursive RNAi of Dicer in which the source of dsRNA decays with first order kinetics to represent dilution of plasmids by cell division, with decay constant δ. All simulations run with γ = 10. (Blue, green, light blue) targeting of reporter only without targeting of RNAi components. (Red, orange, yellow) targeting of Dicer along with the reporter. Decay of dsRNA source leads to transient reduction in reporter that restores at a rate dependent on decay rate of source. Rapid decay of dsRNA source leads to less difference between recursive and non-recursive RNAi (compare δ = 1, where almost no difference is seen when Dicer is also targeted, with a tenfold slower decay rate δ = 0.1, where Dicer targeting clearly increases expression level during transient knockdown). (B) Dependence of restoration on RNAi efficacy parameter γ for different rates of dsRNA source (plasmid) decay.

This situation was modeled as described in Materials and Methods, with results plotted in Figure 9. The results show that introduction of a decay process for the dsRNA source leads to a transient knockdown that eventually returns to baseline expression of the reporter. For slow rates of decay, significant restoration can still be seen with recursive RNAi, but for very fast decay, the restoration becomes negligible. Transient transfection does not, however alter the basic conclusion that increased RNAi efficacy γ leads to decreased restoration. As plotted in Figure 9B, for all rates of decay that were modeled, after increasing γ past an optimum restoration value in the range 1–5, further increasing γ decreases restoration. Thus the basic conclusion that increased RNAi efficacy leads to decreased effectiveness of recursive RNAi is predicted to still hold in transient transfection experiments, although the results also indicate that if the transfection is too transient, restoration might not be detectable in any case.

## Discussion

This report uses a mathematical model to predict the steady-state levels of reporter gene expression in recursive RNAi experiments. This model indicates that recursive RNAi is indeed possible, but that the level of restoration of a reporter gene, and therefore the ability to observe the effect of restoration, depends on the intrinsic efficacy of RNAi knockdown. Systems with more complete RNAi mediated knockdown are predicted to be less susceptible to RNAi. For screens in which the goal is simply to determine whether or not restoration has occurred in order to identify new RNAi components, the level of restoration only needs to be large enough relative to the measurement noise so that a reliable detection can be made. A much more stringent application is when recursive RNAi is used to restore expression of a gene previously inactivated by RNAi, as has been demonstrated in C. elegans [15]. For such switch-off/switch-on applications of recursive RNAi, the level of restoration needs to be sufficiently high to restore essentially wild-type levels of gene function. Restoration of the targeted gene to fully wild-type levels would correspond to a restoration ratio R = 1, which according to Figure 4 is impossible to attain. In many cases, for example genes that are not haplo-insufficient, it may not be necessary to restore gene expression levels all the way to wild-type to rescue the phenotype. However, the results of the model suggest that in many cases, even a more moderate restoration, say to one half or one quarter normal expression levels, would also not be possible if the efficacy of RNAi-mediated knockdown in the organism is too high. One could, in such cases, conduct the experiment in a mutant background with a partial defect in one or more components of the RNAi machinery, so that the value of γ is reduced enough to allow a high level of restoration. Of course, this would entail a design tradeoff because decreased γ would lead to less repression during the switch-off phase of the experiment. In practice, the value of γ might need to be tuned quite carefully to achieve desired results. Moreover, genetic manipulation of the RNAi machinery may lead to undesirable side-effects due to alteration of endogenous small RNA mediated regulatory pathways. A preferable strategy, therefore, may be to carefully tune the relative targeting efficiency [22]–[28] of the reporter versus the RNAi component, so as to reduce the efficacy of targeting of the reporter, which as shown in Figure 6 can produce improved restoration. It is also worth pointing out that inducible systems for turning on and off production of siRNA have been demonstrated [36]–[38]. Recursive-RNAi based switch-off/switch-on has only been documented in nematodes where RNAi constructs can be easily introduced by soaking or feeding, and may be much harder in other types of animals, representing a distinct advantage for inducible systems. Overall, it remains to be seen whether switch-on experiments using recursive RNAi would have any advantages over these chemically inducible approaches. Switch-on by RNAi of RISC components might yield faster dynamics as it would not be limited by the degradation or dilution rate of the siRNA molecules.

In comparing the predictions of the model to experimentally measured levels of reporter gene restoration (see above), it was found that published values for the degree of restoration seen when Argonaute-2 is targeted are much higher than predicted by the model. This does not represent a discrepancy between the model and the data so much as a discrepancy between the experimentally observed behavior of Argonaute-2 and other RNAi components. Indeed, dramatically higher levels of reporter restoration have consistently been reported for Argonaute-2 compared to other RNAi components including Dcr-1, Dcr-2, R2D2, Tudor-SN, FMRp, Drosha, Aubergine, and Piwi [11],[39],[40]. The fact that this protein seems to consistently show a distinctly different behavior in recursive RNAi experiments compared to all other known RNAi components [11] suggests that Argonaute-2 acts somehow differently from the RNAi components described within the model. Perhaps Argonaute-2 might be involved within additional control loops not included in the present model. Consistent with the notion that Ago-2 is somehow unique in its functions and interactions, it has recently been reported that Ago-2 depletion has a distinct and specific effect on RNAi competition that is not seen when other RNAi components are targeted [40]. These considerations suggest that the model used here, in its present form, must not fully represent the range of behavior of Argonaute-2.

The results of Equation 4 indicate that by some measures, the RNAi system may operate more reliably when operated in a closed-loop recursive mode. This result, together with the main result that the susceptibility of the RNAi machinery is to inhibition by RNAi, indicates that the RNAi pathway can demonstrate interesting properties when operated in a closed-loop “recursive” mode, even when represented by a fairly simple model. The favorable comparison with published levels of restoration versus efficacy suggests that the model may have predictive value. Other models of the RNAi pathway have previously been developed which model the system at varying levels of complexity [41]–[44], and it would be interesting to see whether these different models give similar predictions when adapted to represent recursive RNAi experiments. It is also feasible to extend the approach described here to an analysis of the dynamic properties of other types of small RNA mediated control systems such as micro-RNA networks.

## Materials and Methods

### Model Description and Assumptions

The RNAi pathway is represented using a model that is somewhat less complex than previous detailed but non-recursive RNAi models [41]–[44] but which encapsulates the main features of the system. The scheme of the model is given in Figure 1 and the parameters are defined in Table 1. Both Dicer and RISC complexes are represented as single proteins even though in reality both are highly elaborate protein complexes. This representation, employed in most other RNAi models [41]–[44] is justified on the grounds that a typical recursive RNAi experiment would only target a single gene and its corresponding protein, and would not affect other proteins in the complex. Consequently, the protein levels of the other proteins can be simply treated by lumping their effect in with the other constants in the equations.

In the following development only proteins specific to one complex or the other will be treated. In reality, some proteins are shared between the two but this analysis will not consider attempts to silence such shared factors by RNAi. The model will also not address the issue of partial redundancy, in which some RNAi machinery components may be present in multiple gene family members, such that complete inactivation of one member would only result in partial loss of RNAi function. Analysis of switching between different Dicer or Ago family members induced by recursive RNAi would be an interesting area for future study.

To model transcription, it is assumed production of new mRNA at a constant rate r_t_ which is approximately the same for all genes in the model. The model assumes that messenger RNA is degraded through a first-order decay with rate constant r_dm_. Translation of mRNA into protein is modeled assuming that protein is synthesized at a rate proportional to the concentration of message, with a rate constant r_x_, and is degraded with a first order decay rate constant r_dp_. Since the rates of mRNA production and degradation are significantly faster than the corresponding rates for proteins ([45]–[47] and references cited in [42]), a quasi-steady state assumption may be invoked such that mRNA concentrations are set to their presumed steady-state value based on the rates of synthesis and degradation, ignoring the transient behavior while approaching this value.

Production of siRNA by Dicer is represented by assuming that the siRNA is produced at a rate proportional to the concentration of Dicer, with an effective rate constant k_catD_. The concentration of dsRNA is not explicitly represented, rather it is assumed to be lumped into k_catD_, and it is taken as a constant thus assuming that dsRNA will not be degraded over time. The latter assumption is most appropriate for systems in which the dsRNA is expressed constitutively within the cell as a small hairpin construct. It is further assumed that siRNA is degraded by a first order decay with rate constant r_ds_. In the simplest form of this model, to be described first, the production of additional dsRNA from targeted message by RNA-dependent RNA polymerase [32]–[34],[42] is not modeled, but the effect of such an enzyme will be considered later in this report.

It is assumed that an siRNA molecule is loaded onto a RISC complex according to a simple first-order binding process with an affinity described by the dissociation constant K_DR_. This assumption implies that the RISC complex is not saturated by siRNA during the modeled experiments. This assumption may not always hold true. It has been shown that when multiple siRNA species are added to a cell or in vitro RNAi system, they can compete with each other [48]–[50], and this is thought to reflect a limited quantity of Ago2 that becomes saturated when too many siRNAs are present [40]. Whether or not RISC/Ago2 becomes saturated will depend on how much siRNA is used, for instance in one vitro study it was found that 100–200 fold more siRNA than normally used was required to show significant competition, suggesting that in the normal experimental regime employed by those workers, RISC was not saturated [48]. In the present model, saturation of RISC binding would imply an excess of siRNA thus rendering the system less sensitive to recursive RNAi targeting of Dicer.

To model degradation of target messages by the RISC complex, it is assumed that a message targeted by an siRNA will be degraded by RISC at a rate equal to the product of the concentration of siRNA-loaded RISC and the concentration of the target message, with a rate constant k_catR_. The linear dependence of RISC complex formation and activity on siRNA and RISC concentrations, including the assumptions of first order binding and lack of saturation, are in agreement with the prior modeling studies of RNAi [41]–[43].

The following analysis of the model will only keep track of proteins whose level will change during the course of an experiment. Proteins that are not affected by the addition of the dsRNAs, will be assumed to have attained their steady state value long before the beginning (τ = 0) of the experiment. They will, therefore, be treated as constants of the model, just as the levels of basic transcriptional and translational machinery are assumed constant in the model. While the model explicitly treats only one protein component of the Dicer or RISC complexes at a time in the analysis, since in a typical recursive RNAi experiment only one protein would be targeted, in fact the model does not in any way place any limits on the number of proteins that may be present in the two complexes. However, the influence of the other proteins is subsumed within the other parameters of the model, and is taken as constant under the assumption that the other proteins in the Dicer and RISC complexes, apart from whichever protein might be targeted by recursive RNAi, do not vary in their expression levels.

The following discussion will refer to the reporter gene as GFP, but would describe any target gene such as luciferase.

The behaviors of components of the RNAi machinery, plus a reporter construct, can be represented as follows in three distinct cases:

### Equations Governing RNAi in Open-Loop and Recursive Configurations

#### Case I. No recursive RNAi

***Sub-case IA. No RNAi of reporter or of RNAi machinery.*** Reporter protein is translated at a constant rate from the corresponding mRNA which is presumed to have reached its own steady-state level given by r_t_/r_dm_, and the protein is degraded with a first order rate constant yielding:
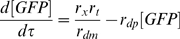
(I.A.1)


***Sub-case IB. RNAi of reporter gene only.*** When RNAi targets the reporter mRNA, we augment Equation I.A.1 with a second mRNA degradation rate reflecting RISC activity:
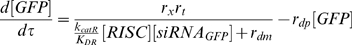
(I.B.1)Equation I.B.1 is derived assuming that the concentration of active siRNA-loaded RISC is at a quasi-steady state found by considering the concentration of RISC protein, the concentration of siRNA, and the dissociation constant describing their interaction. This quasi-steady state assumption allows us to avoid explicitly modeling the rate of formation of siRNA loaded RISC, and the same assumption has been employed in other models of RNAi [43].

The siRNA targeting the reporter is formed by the action of Dicer and is degraded with first-order kinetics yielding:
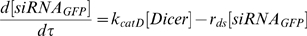
(I.B.2)Proteins not targeted by RNAi are present at a steady-state level as follows:
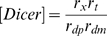
(I.B.3)

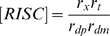
(I.B.4)


#### Case II. RNAi targeting Dicer and a reporter gene



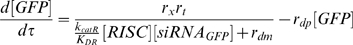
(II.1)

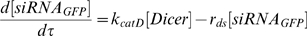
(II.2)In this case Dicer is also a target and so its production is described in a similar form to that used for the reporter gene, yielding:
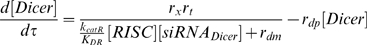
(II.3)

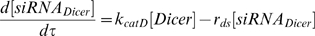
(II.4)Because RISC is not targeted along with Dicer, it remains at its steady-state value:
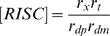
(II.5)


#### Case III. RNAi targeting RISC and a reporter gene



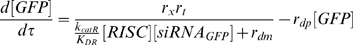
(III.1)

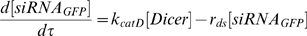
(III.2)In this case, RISC, rather than Dicer, has its production term modified to reflect message degradation by RNAi as follows:
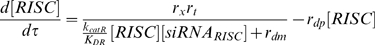
(III.3)

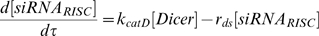
(III.4)

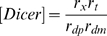
(III.5)


### Rescaling and Simplifying Substitutions

In order to simplify the equations representing the model, time, protein concentration, and siRNA concentration are rescaled as follows, representing the rescaled concentrations with capital letters:
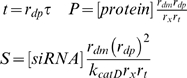
To simplify the resulting expressions, the following lumped parameters are defined as combinations of the detailed parameters of the model summarized in Table 1:
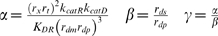



### Fixed Points and Stability for the Individual Cases

#### Case IA

Let G represent the protein level for the reporter (e.g. GFP).

First rescale time as follows:
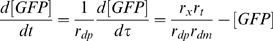
Next, rescale reporter protein concentration as follows:
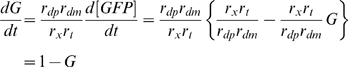
The steady-state solution is:
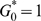
Because of the way all protein concentrations are rescaled, steady-state concentration of any protein not targeted by RNAi is always 1.

#### Case IB

Let G represent the rescaled reporter protein level and W represent the rescaled siRNA level directed against the reporter gene. Rescaling time and substituting the steady-state Dicer concentration yields:
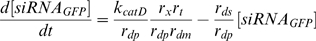
Rescaling siRNA concentrations yields:
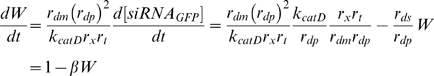
Rescaling time and then protein concentration as above yields:
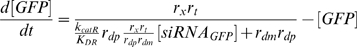


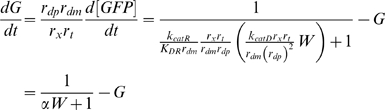
yielding the planar system:
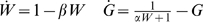
which has steady state solution:
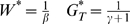
The steady-state reporter protein level under these conditions is denoted by the subscript T to indicate that the reporter is targeted by RNAi. The steady state value of G_T_ demonstrates the significance of the parameter gamma as an indicator of the efficacy of RNAi. Larger gamma means that the level of reporter protein is reduced to a greater extent relative to its steady-state value in the absence of RNAi (case IA) when G_0_* = 1. Taking the ratio of the steady-state GFP levels with (G_T_) and without (G_o_) RNAi of GFP yields Equation 1 given in the Results section, which specifies the fold of knockdown of the targeted gene in terms of the RNAi efficacy parameter γ.

Linearizing this system around G_T_*,W* yields the Jacobian matrix:
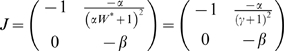
For which Det(J) = β is strictly positive and Tr(J) = −1−β is strictly negative, hence the fixed point is stable for the linearized system. The eigenvalues of J are equal to −1 and −β, hence the fixed point is hyperbolic so the fixed point is locally stable for the nonlinear system as well.

The divergence for the nonlinear system 
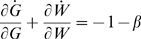
 is strictly negative for all values of G and W thus satisfying Bendixson's criterion [51] ruling out the existence of any closed orbits. Since the system is planar, the existence of a unique locally stable fixed point together with the lack of any closed orbits implies that the fixed point must be globally attracting.

#### Case II

**RNAi of Dicer plus a reporter gene.** Let X represent Dicer protein and Z the siRNA directed against the Dicer gene, while G and W will represent the protein and siRNA for the reporter gene as in the previous case. The substitutions employed above yield the system:
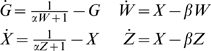
Since G and W have no effect on X and Z, it is sufficient to analyze just the planar system (X,Z):
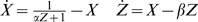
This system has a single physically realizable fixed-point:
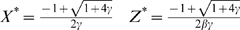
It is easily shown that this fixed point is stable and globally attracting. Taking the ratio of Dicer levels when Dicer is targeted by RNAi (X = X*) versus when Dicer is not targeted (X = 1) yields Equation 2 given in the results section, which expresses the fold knockdown of Dicer during recursive RNAi.

When X and Z reach steady state, the steady-state levels of the reporter-targeting siRNA and reporter protein (denoted G_TD_ to signify simultaneous targeting of Dicer) are:
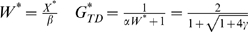
This value G_TD_ of GFP during recursive RNAi of Dicer gives Equation 3 of the Results section. It is easily shown that the level of GFP and the level of Dicer are strictly equal to each other during recursive RNAi of Dicer, not only at steady state but also transiently.

#### Case III

**RNAi of a RISC complex specific gene product plus the reporter gene.** Starting with the equations listed above for this case and making the usual rescaling operations yields the following set of equations in which Y represents the rescaled level of RISC protein and Z the level of the corresponding siRNA:
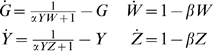
Analyzing the RNAi machinery itself (Y,Z) and ignoring the reporter gene yields the planar system:
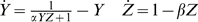
which has one physically realizable fixed point:
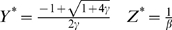
This fixed point is easily shown to be stable and attracting for Y≥0, Z≥0.

The level of the reporter can be determined once the system has reached steady state:
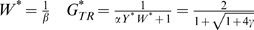
Where the subscript on G denotes the case that the reporter is targeted along with RISC.

### Figures of Merit for Optimization of Recursive RNAi Experiments

First consider the relative susceptibility of Dicer and RISC proteins to downregulation by RNAi compared with a generic reporter protein that is not a component of the RNAi machinery. In other words, is recursive RNAi more or less effective compared with open-loop RNAi?

The relative susceptibilities S_D_ and S_R_ of Dicer and RISC, respectively, relative to the reporter gene, are defined as:
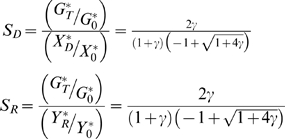
it is obvious by inspection that the relative susceptibility of the two components of the RNAi machinery will decrease relative to the reporter gene as the efficacy of RNAi increases (as judged by the parameter gamma). So as RNAi efficacy increases, RNAi genes become increasingly resistant to RNAi.

In a typical recursive RNAi experiment, usually only the reporter protein level is measured, rather than the level of Dicer or RISC proteins. A candidate gene is scored in screens as being involved in RNAi if dsRNA directed against the gene results in a restoration of reporter gene activity back to control levels. In other words, if one monitors the reporter protein level, when it is targeted by RNAi the level will drop, and if a component of the RNAi machinery is also targeted, the level of the reporter will rise back up towards its level seen when no RNAi is performed.

One way to quantify this restoration effect is to measure the ratio of recovery after recursive RNAi knockdown to the level of knockdown relative to control. This is expressed by the relative restoration ratios R_D_ and R_R_ which can be defined for the two cases RNAi of Dicer and RNAi of RISC, respectively, as follows:
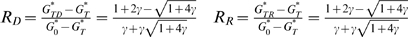



For a switch-off/switch-on experiment using Dicer, for example, one would want G_TD_≈G_0_, which in turn would require that R_D_≈1. In fact, R_D_ is maximal when γ = 2, and its maximum value is only 0.25. It is thus not possible to restore gene expression back to fully normal levels, but only at most one quarter of the way back to normal levels from the level of maximum knockdown prior to “switch on”.

As an alternative to these ratios, one may be more interested in the absolute difference in expression levels in the two conditions of knockdown versus knockdown in the presence of recursive RNAi. This difference ultimately determines the detectability of gene restoration when compared with the standard deviation of measurement of expression levels in the two states. The increase in expression levels, in units normalized to the control expression level of the reporter gene, is given by:
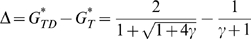



### Differential Efficiency of Targeting between Reporter and Dicer

Suppose that due to difference in targeting sequences, siRNA inhibition of GFP (or whatever gene of interest is being knocked down) is either more or less efficient than siRNA inhibition of Dicer in a recursive RNAi experiment. This effect can be represented in the model above as a difference in catalytic efficiency of siRNA-loaded RISC. This can be represented by a parameter *ε* such that if k_catR_ is the catalytic rate constant of RISC when acting on Dicer, the catalytic rate constant of RISC when acting on GFP would be ε*k_catR_. In this case the only change to the systems described above will be to the differential equations representing the rate of change of GFP level, as follows:
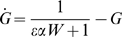
using this modified equation to solve for the steady-state GFP level yields:
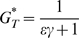
for the non-recursive case, and
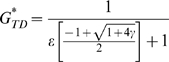
These two expressions were used to plot the predicted expression during recursive RNAi with differential targeting in Figure 6.

The relative restoration ratio for the GFP target before and after recursive RNAi of Dicer is then given, as a function of the relative targeting efficiency of GFP, by the equation:
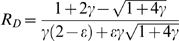



#### Sensitivity to parameter variation

This section will consider the sensitivity of Dicer protein level to fluctuations in the rate of Dicer translation. In the open-loop configuration, that is, where Dicer is not itself a target of RNAi, the steady-state concentration of Dicer protein is easily found from the equations above to be:
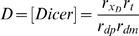
Where r_xD_ specifies the translation rate of Dicer, which is the quantity that will be allowed to fluctuate. All other parameters will be assumed constant. The open-loop sensitivity S_open_ is defined as the magnitude of change in Dicer produced by a small change in r_xD_:
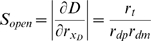
For the closed-loop configuration produced by recursive RNAi targeting Dicer the steady-state concentration of Dicer protein is:
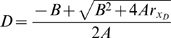
where we have defined:
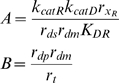
Hence the sensitivity in this case is:
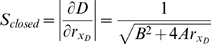
Equation 4 in the Results section follows from the ratio of the sensitivity in the closed versus open loop configuration and recognizing that AC/B^2^ = γ.

#### Modeling effect of RdRP-mediated amplification

The effect of siRNA amplification [32]–[34] can be incorporated into the model by adding a term to the differential equations describing the change in siRNA levels in order to represent a secondary pathway for siRNA production. Based on the model for amplification in C. elegans [32]–[34] the rate of production of secondary siRNA should be proportional to the product of the concentration of siRNA-loaded RISC and the concentration of targeted mRNA. For case IB, in which the reporter is targeted but none of the RNAi machinery is targeted, the addition of this extra term yields a new expression for the normalized siRNA level W as follows:
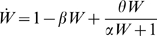
Where θ is a constant of proportionality that determines the number of siRNA molecules produced per RISC-targeted mRNA. The numerator of the new term reflects the assumption that secondary siRNA production is proportional to the concentration of siRNA-loaded RISC, and assumes that the concentration of RISC is unaffected in the experiment and hence equal to one in normalized units. The denominator arises from the steady state mRNA concentration in normalized units as discussed above when deriving the protein production rate term. All necessary normalizing constants are included in the single parameter θ. Larger values of this parameter imply more efficient secondary amplification. The differential equation describing protein production and degradation is unaffected by the addition of the amplification pathway and remains the same as that given above under case IB.

For case II, in which Dicer is targeted by recursive RNAi, the equations describing dicer and reporter protein are unaffected, but the equations describing the two siRNA species Z and W, which are the siRNAs targeting Dicer and GFP, become:
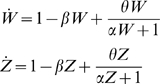
The plots in Figure 7 were generated by numerical integration using these equations plus the equations for dicer protein and GFP given above and assuming β = 1. We note that in the model for amplification currently thought to apply in C. elegans, Dicer plays no direct role in production of secondary siRNA molecules. In plants where Dicer is though to generate the secondary siRNA from cleavage of dsRNA made from targeted message, the resulting dynamics might become more complicated because the level of Dicer protein would now appear in the production term for Dicer-directed siRNA. Analysis of behavior in more complicated systems like this will be an interesting area for future study.

#### Modeling non-essential components

The effect of targeting components that make a partial contribution to RNAi effectiveness is modeled by positing a new parameter rho that determines the extent to which a component is required for RNAi. Here, the specific case of a component that contributes partially to Dicer activity is modeled, although a similar development can be shown for a non-essential component of RISC activity. For a partial contribution to Dicer activity, the rate of siRNA production, normally set equal to the normalized concentration X of Dicer (see above) is replaced with ρX+(1−ρ), which is equal to X when rho is 1 indicating a central component, and is 0 when rho is zero, indicating a component that does not contribute to Dicer activity at all. As before X still represents the protein level of the component that is targeted. For recursive RNAi of such a component, the new system of equations is obtained:
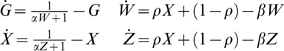
with steady state solution for reporter expression level:
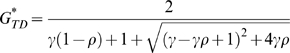



This expression was used to plot Figure 8. It is easily verified that for ρ = 0 this expression matches the reporter level in the absence of recursive RNAi, as expected if the component has no effect on RNAi activity. For ρ = 1, the expression becomes identical to that derived in Case II above, i.e. for a component that is absolutely required for Dicer activity. It can be shown that the expression level of the reporter is maximum when ρ = 1, indicating that recursive targeting of an essential component will give greater restoration than targeting of a non-essential component.

#### Modeling transient transfection

To model transient transfection, the same numerical simulation employed above to model transient behavior of recursive RNAi was modified to include a parameter φ proportional to the concentration of dsRNA expressing plasmid. This parameter was initialized to a value of 1 and allowed to decay with first order rate constant δ. The rate of production of siRNA was changed from X to φX, reflecting our assumption that siRNA production would be proportional to the plasmid concentration as well as to the concentration of Dicer complex. This modification was applied to both open-loop and recursive RNAi of the reporter and the results plotted in Figure 9A. In order to generate Figure 9B, it was necessary to choose a transient time-point to assess restoration, since due to decay of the plasmid, all cases eventually return to full expression once the plasmid decays completely, making restoration impossible to assess at the steady state. The time point at which reporter expression was minimized in the open-loop case was chosen as the reference time point, and restoration was calculated at that point using the equation for restoration described above. These results were calculated for a range of γ and δ and plotted in Figure 9B.
